# Supplementary material for: Codon Usage for Genetic Diversity, and Evolutionary Dynamics of Novel Porcine Parvoviruses 2 through 7 (PPV2–PPV7)
Source: Viruses. 2022 Jan 18;14(2):170. doi: 10.3390/v14020170 (PMC8876854; doi:10.3390/v14020170)
Supplement: Supplementary file 1 [file viruses-14-00170-s001.zip › Supplementary Figure S1.pdf]

Supplementary Figure S1.

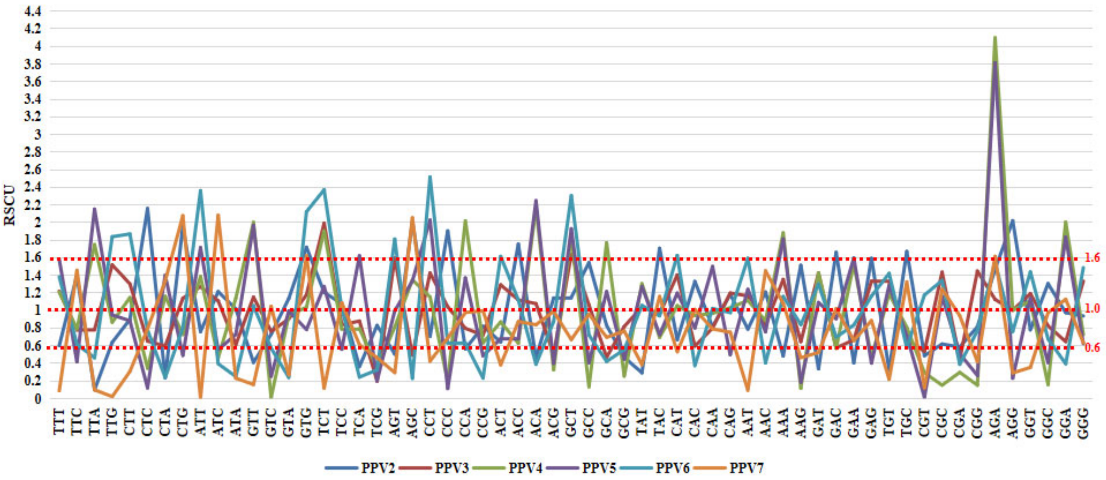

Figure S1. Comparative analysis of relative synonymous codon usage (RSCU) values of PPV subtypes. The colors of PPV2, PPV3, PPV4, PPV5, PPV6 and PPV7 are dark blue, brown, green, purple, light blue and yellow, respectively.
