# Supplementary material for: Codon Usage for Genetic Diversity, and Evolutionary Dynamics of Novel Porcine Parvoviruses 2 through 7 (PPV2–PPV7)
Source: Viruses. 2022 Jan 18;14(2):170. doi: 10.3390/v14020170 (PMC8876854; doi:10.3390/v14020170)
Supplement: Supplementary file 1 [file viruses-14-00170-s001.zip › Supplementary Figure S2.pdf]

Supplementary Figure S2.

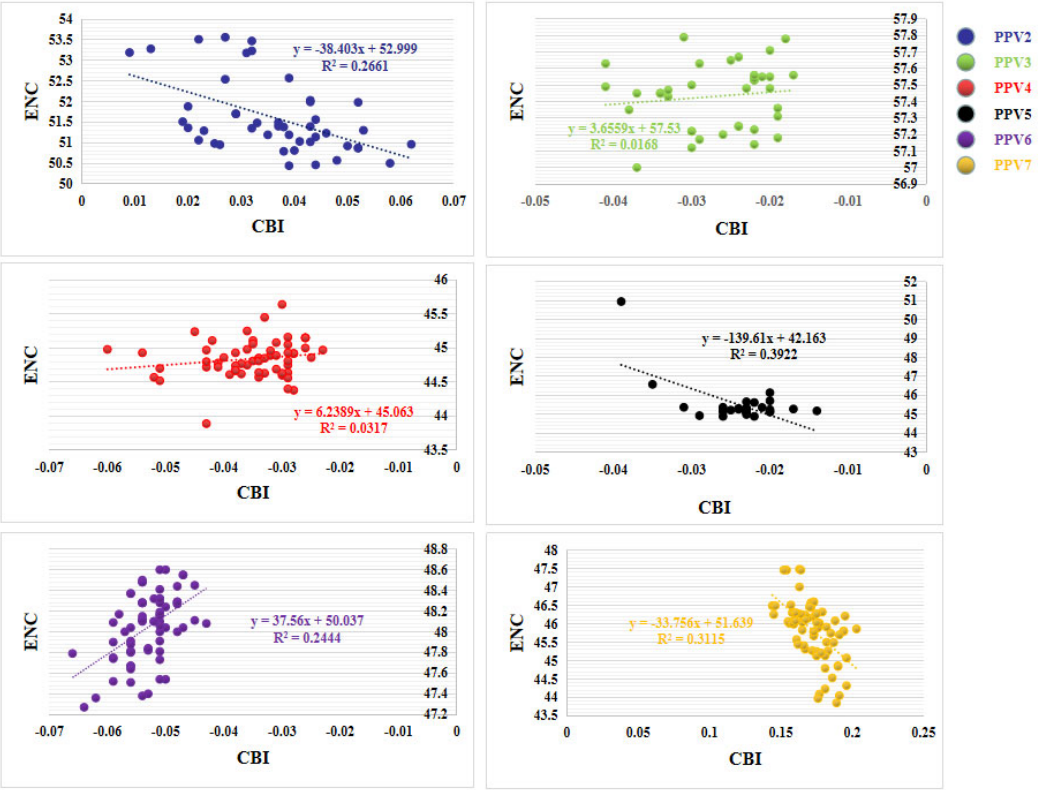

Figure S2. For the target host's own genes, CBI and ENC values have a good correlation, which more clearly reflects the expression of foreign genes in the target host.
