# Supplementary material for: Codon Usage for Genetic Diversity, and Evolutionary Dynamics of Novel Porcine Parvoviruses 2 through 7 (PPV2–PPV7)
Source: Viruses. 2022 Jan 18;14(2):170. doi: 10.3390/v14020170 (PMC8876854; doi:10.3390/v14020170)
Supplement: Supplementary file 1 [file viruses-14-00170-s001.zip › Supplementary materials.pdf]

S1. Codon Usage Index

S1.1. Effective Number of Codons (ENC) Analysis

The ENC value ranges from 20 (in the case of extreme deviations, where only one codon is used for each amino acid) to 61 (when the possibility of using alternative synonymous codons is the same), a higher ENC value means a lower Codon usage bias. Conversely, the smaller the ENC value, the higher the expression level of the corresponding endogenous gene. Therefore, it provides an intuitive and meaningful way to measure the degree of gene codon preference. The expected ENC is calculated as follows:

$$\mathrm{ENC} = 2 + \frac{9}{F_2} + \frac{1}{F_3} + \frac{5}{F_4} + \frac{3}{F_6} \tag{S1}$$

$$F = \frac{n\sum_{i=1}^{k} p_i^2 - 1}{n-1} \qquad n > 1 \quad p_i = \frac{n_i}{n} \tag{S2}$$

F_i (i = 2,3,4,6) is the average of the F_i value for i-fold degenerate amino acids **(S1)**. n represents the total number of codons observed for the amino acid, k is the number of synonymous codons, and pi is the frequency of the i-th codon (ni/n), Where represents the total number of codons used in the gene, and 'i' is the number of synonymous codons **(S2)**.

S1.2. Relative Synonymous Codon Usage Analysis (RSCU)

The calculation method of this value is as follows: In the formula, xij is the number of occurrences of the j codon encoding the i-th amino acid, and ni is the number of synonymous codons encoding the i amino acid. Values of RSCU greater than 1.6 and less than 0.6 are considered "over-represented" and "under-represented", respectively (Wong et al., 2010).

$$\mathrm{RSCU}_{ij} = \frac{x_{ij}}{\frac{1}{n_i}\sum_{j=1}^{n_i} x_{ij}}$$

.

S1.3. Relative Codon Deoptimization Index (RCDI)

The RCDI/eRCDI server calculates RCDI through the equation: 1. CiFa (relative frequency of codon i of a specific amino acid in the test sequence); 2. CiFh (relative frequency of codon i of a specific amino acid in the reference sequence); 3. Ni (the number of occurrences of codon i in the test sequence); 4. N (the total number of codons in the test sequence). RCDI ranges from 1 (the codon usage of the test sequence is fully optimized to the codon usage of the reference genome) to N (increases with the deoptimization of the test sequence).

$$RCDI = \sum_{i=1}^{61} \frac{\frac{CiFa}{CiFh} Ni}{N}$$

S1.4. Codon Adaptation Index (CAI) Analysis

w_{ij} (The relative adaptiveness of a codon): The relative adaptiveness of codons. In the following formula, $RSCU_{imax}$ and X_{imax} respectively refer to the RSCU value and X value of the most frequently used codon that encodes the i-th amino acid.

$$w_{ij} = \frac{\mathrm{RSCU}_{ij}}{\mathrm{RSCU}_{i\max}} = \frac{x_{ij}}{x_{i\max}}$$

Codon Adaptation Index (CAI) is a measure of the bias of synonymous codon usage in DNA or RNA sequences. It can be used to predict gene expression levels, assess the adaptability of viral genes to its host, and compare codon usage in different organisms (Sharp and Li, 1987). Use CAIcal server (http://genomes.urv.cat/CAIcal/RCDI/) to estimate. L refers to the number of codons used in the gene.

$$\mathrm{CAI} = \left(\prod_{K=1}^{L} w_K \right)^{\frac{1}{L}}$$

S1.5. Frequency of Optimal Codons (FOP) Analysis

FOP The value range is between 0 and 1. “1” means that only the optimal codon is used, and “0” means that no optimal codon is used, and the determination of the optimal codon requires a set of gene sequences and corresponding expression information.

$$\mathrm{FOP}(g) = \sum_i f_{aa(i)}(g)\mathrm{RSCU}_i(g)$$

$$f_{aa(i)} = n_{aa(i)}(g) \ / N$$

Where ni(g) is the number of codon i in gene g, and N is the total number of codons in g, sum all the best codons. Where naa(i)(g) is the number of amino acids coded by i in g. The second multiplier is simply the relative synonymous codon usage or RSCU of codon i in gene g

S1.6. Codon Bias Index (CBI) Analysis

N_{opt} represents the sum of the number of occurrences of superior codons in the gene; N_{ran} represents the sum of the number of occurrences of superior codons when the amino acid sequence remains unchanged; N_{tot} represents the sum of the number of times the amino acid corresponding to the superior codon appears in the gene. Calculated as follows:

$$\mathrm{CBI} = \frac{N_{\mathrm{opt}} = N_{\mathrm{ran}}}{N_{\mathrm{tot}} - N_{\mathrm{ran}}}$$

References

1. Sharp, P.M., Li, W.H. The codon Adaptation Index--a measure of directional synonymous codon usage bias, and its potential applications. *Nucleic Acids Res.* **1987**, *15*, 1281-1295.
2. Wong, E.H., Smith, D.K., Rabadan, R., Peiris, M., Poon, L.L. Codon usage bias and the evolution of influenza A viruses. Codon Usage Biases of Influenza Virus. BMC *Evol. Biol.* **2010**, *10*, 253.
