# Supplementary material for: Codon Usage for Genetic Diversity, and Evolutionary Dynamics of Novel Porcine Parvoviruses 2 through 7 (PPV2–PPV7)
Source: Viruses. 2022 Jan 18;14(2):170. doi: 10.3390/v14020170 (PMC8876854; doi:10.3390/v14020170)
Supplement: Supplementary file 1 [file viruses-14-00170-s001.zip › Supplementary Table S1.pdf]

Table S1. Reference strains information of PPV2.

Strsins	GenBank accession NO.	Year	Area
BR/GO/ion-09	KM926355.1	2011	Brazil
US-135	JX101461.1	2012	USA
US-523	JX101462.1	2012	USA
215	KP245947.1	2015	China
GX-1-NS1	MZ892964.1	2017	China
GX-15-NS1	MZ892965.1	2017	China
GX-16-NS1	MZ892966.1’	2017	China
GX-18-NS1	MZ892967.1	2017	China
AH01-C1	MK378157.1	2019	China
BJ01-C1	MK378158.1	2019	China
CQ01-C1	MK378159.1	2019	China
FJ01-C1	MK378160.1	2019	China
GD01_C1	MK378162.1	2019	China
GD02-C1	MK378163.1	2019	China
GX04-C1	MK378172.1	2019	China
GX03-C1	MK378170.1	2019	China
GZ02-C1	MK378179.1	2019	China
GZ03-C1	MK378180.1	2019	China
HeB01-C1	MK378183.1	2019	China
HeB03-C1	MK378187.1	2019	China
HeB04-C1	MK378188.1	2019	China
HeN01-C1	MK378189.1	2019	China
HLJ01-C1	MK378190.1	2019	China
HuN01-C1	MK378195.1	2019	China
HuN02-C1	MK378197.1	2019	China
JS01-C1	MK378200.1	2019	China
JX01-C1	MK378201.1	2019	China
LN02-C1	MK378206.1	2019	China
NM01-C1	MK378207.1	2019	China
NM02-C1	MK378208.1	2019	China
NX01-C1	MK378209.1	2019	China
NX02-C1	MK378211.1	2019	China
SC02_C1	MK378213.1	2019	China
SD01-C1	MK378214.1	2019	China
SD02-C1	MK378215.1	2019	China
SH01-C1	MK378217.1	2019	China
SX01-C1	MK378218.1	2019	China
SX02-C1	MK378219.1	2019	China

TJ01-C1	MK378220.1	2019	China
ZJ01-C1	MK378224.1	2019	China
JX14	MK092400.1	2020	China
JX19	MK092401.1	2020	China
JX26	MK092402.1	2020	China
180405-97-PPV2	MH921914.1	2020	Korea
MW051675.1	P1-OK/USA	2021	Korea
