# Supplementary material for: Codon Usage for Genetic Diversity, and Evolutionary Dynamics of Novel Porcine Parvoviruses 2 through 7 (PPV2–PPV7)
Source: Viruses. 2022 Jan 18;14(2):170. doi: 10.3390/v14020170 (PMC8876854; doi:10.3390/v14020170)
Supplement: Supplementary file 1 [file viruses-14-00170-s001.zip › Supplementary Table S2.pdf]

Table S2. Reference strains information of PPV3.

Strsins	GenBank accession NO.	Year	Area
IA38835/1998	KF561853.1	1998	USA
IA40895/1998	KF561854.1	1998	USA
PPV3-03	MK649762.1	2002	United Kingdom
PPV3-02	MK649763.1	2002	United Kingdom
PPV3-01	MK649761.1	2004	United Kingdom
IIISRB	KC701328.1	2007	Serbia
ISRB	KC701332.1	2007	Serbia
IISRB	KC701329.1	2008	Serbia
IVSRB	KC701321.1	2008	Serbia
302HU	KC701327.1	2008	Hungary
340HU	KC701326.1	2008	Hungary
359HU	KC701325.1	2008	Hungary
360HU	KC701319.1	2008	Hungary
643CRO	KC701315.1	2008	Croatia
655CRO	KC701320.1	2008	Croatia
VISRB	KC701330.1	2008	Serbia
676CRO	KC701323.1	2009	Croatia
687CRO	KC701318.1	2010	Croatia
705CRO	KC701317.1	2010	Croatia
MK649764.1	16	2010	United Kingdom
MK649765.1	17	2010	United Kingdom
MK649766.1	18	2010	United Kingdom
MK649767.1	19	2010	United Kingdom
MK649768.1	21	2010	United Kingdom
MK649769.1	22	2010	United Kingdom
722CRO	KC701322.1	2011	Croatia
136PL	KC701331.1	2011	Poland
VIIISRB	KC701316.1	2011	Serbia
CQ01	MK378226.1	2017	China
GX02	MK378230.1	2017	China
GZ03	MK378232.1	2017	China
GZ04	MK378233.1	2017	China
HeN01	MK378234.1	2017	China
LN02	MK378238.1	2017	China
NX01	MK378239.1	2017	China
NX02	MK378240.1	2017	China
SH01	MK378244.1	2017	China
E1	MW030463.1	2018	China

IZS-16	MH884552.1	2019	Italy
