# Supplementary material for: Codon Usage for Genetic Diversity, and Evolutionary Dynamics of Novel Porcine Parvoviruses 2 through 7 (PPV2–PPV7)
Source: Viruses. 2022 Jan 18;14(2):170. doi: 10.3390/v14020170 (PMC8876854; doi:10.3390/v14020170)
Supplement: Supplementary file 1 [file viruses-14-00170-s001.zip › Supplementary Table S3.pdf]

Table S3. Reference strains information of PPV4.

Strsins	GenBank accession NO.	Year	Area
PPV4-01	MK609918.1	2002	United Kingdom
VIIISRB	KC701338.1	2006	Serbia
296HU	KC701339.1	2007	Hungary
40HU	KC701341.1	2007	Hungary
285HU	KC701342.1	2007	Hungary
263HU	KC701349.1	2007	Hungary
III32SRB	KC701351.1	2007	Serbia
VSRB	KC701353.1	2007	Serbia
IISRB	KC701354.1	2007	Serbia
317HU	KC701340.1	2008	Hungary
299HU	KC701343.1	2008	_Hungary
654CRO	KC701344.1	2008	Croatia
IVSRB	KC701345.1	2008	Serbia
S-145-2HU	KC701346.1	2008	Hungary
PPV4.complete genome	KY586146.1	2008	Brazil
HEN0922-5400	GU978964.1	2009	China
HEN0922-5645	GU978965.1	2009	China
JS0918-5598	GU978966.2	2009	China
JS0910-5644	GU978967.1	2009	China
JS0910-5400	GU978968.1	2009	China
JS0918a	HM031134.1	2009	China
JS0918b	HM031135.1	2009	China
66PL	KC701333.1	2011	Poland
97PL	KC701334.1	2011	Poland
107PL	KC701335.1	2011	Poland
150PL	KC701336.1	2011	Poland
85G12PL	KC701337.1	2011	Poland
33PL	KC701347.1	2011	Poland
21SRB	KC701348.1	2011	Serbia
185PL	KC701350.1	2011	Poland
21PL	KC701352.1	2011	Poland
99PL	KC701355.1	2011	Poland
JX15	MK092418.1	2015	China
JX28	MK092419.1	2015	China
JX24	MK092420.1	2015	China
HB3	KP245953.1	2017	China
171206-10-PPV4	MH921902.1	2017	Korea
CQ01	MK378247.1	2017	China

GD01	MK378249.1	2017	China
GX05	MK378255.1	2017	China
GZ02	MK378257.1	2017	China
HeB01	MK378260.1	2017	China
HeB03	MK378262.1	2017	China
HeB04	MK378263.1	2017	China
HeN01	MK378264.1	2017	China
HuN01	MK378268.1	2017	China
HuN02	MK378269.1	2017	China
JL01	MK378270.1	2017	China
LN01	MK378273.1	2017	China
NM02	MK378276.1	2017	China
NX02	MK378278.1	2017	China
SX01	MK378286.1	2017	China
180119-2-PPV4	MH921910.1	2018	Korea
180119-23-PPV4	MH921911.1	2018	Korea
180605-30-PPV4	MH921915.1	2018	Korea
MK649769.1P1_OK/USA	MW073110.1	2018	USA
QNi17	MT434667.1	2019	Vietnam
QT02	MT434668.1	2019	Vietnam
QT20	MT434669.1	2019	Vietnam
