# Supplementary material for: Codon Usage for Genetic Diversity, and Evolutionary Dynamics of Novel Porcine Parvoviruses 2 through 7 (PPV2–PPV7)
Source: Viruses. 2022 Jan 18;14(2):170. doi: 10.3390/v14020170 (PMC8876854; doi:10.3390/v14020170)
Supplement: Supplementary file 1 [file viruses-14-00170-s001.zip › Supplementary Table S4.pdf]

Table S4. Reference strains information of PPV5.

Strsins	GenBank accession NO.	Year	Area
MI216	JX896318.1	2011	USA
IN273	JX896319.1	2011	USA
IA469	JX896320.1	2011	USA
ND564	JX896322.1	2011	USA
P13-9	KX352455.1	2016	Poland
P13-10	KX352456.1	2016	Poland
P12-1	KX352457.1	2016	Poland
K17-1	KX352458.1	2016	Poland
171206-29-PPV5	MH921904.1	2017	Korea
171220-17-PPV5	MH921908.1	2017	Korea
AH01	MK378291.1	2017	China
BJ01	MK378292.1	2017	China
CQ01	MK378293.1	2017	China
FJ01	MK378295.1	2017	China
GD02	MK378298.1	2017	China
GZ01	MK378305.1	2017	China
GZ03	MK378307.1	2017	China
GZ04	MK378308.1	2017	China
HeB01	MK378309.1	2017	China
HeB03	MK378312.1	2017	China
HuN01	MK378325.1	2017	China
LN01	MK378332.1	2017	China
NM02	MK378336.1	2017	China
NX01	MK378337.1	2017	China
NX02	MK378338.1	2017	China
SD01	MK378341.1	2017	China
SD02	MK378342.1	2017	China
SX01	MK378344.1	2017	China
TJ01	MK378346.1	2017	China
YN01	MK378347.1	2017	China
ZJ01	MK378349.1	2017	China
180119-23-PPV5	MH921912.1	2018	Korea
PPV5-P2 OK/USA	MT671979.1	2018	Brasil
P1_OK/USA	MW051674.1	2018	USA
P2_OK/USA	MW051673.1	2019	USA
