# Supplementary material for: Codon Usage for Genetic Diversity, and Evolutionary Dynamics of Novel Porcine Parvoviruses 2 through 7 (PPV2–PPV7)
Source: Viruses. 2022 Jan 18;14(2):170. doi: 10.3390/v14020170 (PMC8876854; doi:10.3390/v14020170)
Supplement: Supplementary file 1 [file viruses-14-00170-s001.zip › Supplementary Table S5.pdf]

Table S5. Reference strains information of PPV6.

Strsins	GenBank accession NO.	Year	Area
BJ	KF999681.1	2013	China
BJ2	KF999682.1	2013	China
JS	KF999683.1	2013	China
SC	KF999684.1	2013	China
TJ	NC_023860.1	2013	China
TJ	KF999685.1	2013	China
GX-1	MZ846220.1	2017	China
GX-15	MZ846221.1	2017	China
GX-30	MZ846219.1	2017	China
GX-65	MZ846222.1	2017	China
170403-49-PPV6	MH921900.1	2018	Korea
70405-1-PPV6	MH921901.1	2018	Korea
171206-11-PPV6	MH921903.1	2018	Korea
171219-40-PPV6	MH921906.1	2018	Korea
171220-11-PPV6	MH921907.1	2018	Korea
171220-44-PPV6	MH921909.1	2018	Korea
180119-25-PPV6	MH921913.1	2018	Korea
AH01	MK378350.1	2018	China
BJ01	MK378351.1	2018	China
CQ01	MK378352.1	2018	China
GD02	MK378355.1	2018	China
GD03	MK378356.1	2018	China
GX03	MK378360.1	2018	China
GX04	MK378361.1	2018	China
GZ01	MK378364.1	2018	China
GZ02	MK378365.1	2018	China
GZ03	MK378366.1	2018	China
GZ04	MK378367.1	2018	China
HeB01	MK378368.1	2018	China
HeB02	MK378369.1	2018	China
HeB04	MK378371.1	2018	China
HeN01	MK378372.1	2018	China
HLJ01	MK378373.1	2018	China
HuB01	MK378374.1	2018	China
HuN01	MK378376.1	2018	China
HuN02	MK378377.1	2018	China
JX01	MK378381.1	2018	China
JX01	MK378382.1	2018	China

LN02	MK378386.1	2018	China
NM01	MK378387.1	2018	China
NM02	MK378388.1	2018	China
NX01	MK378389.1	2018	China
NX02	MK378390.1	2018	China
SC02	MK378392.1	2018	China
SD01	MK378393.1	2018	China
SH01	MK378396.1	2018	China
SX01	MK378397.1	2018	China
SX02	MK378399.1	2018	China
TJ01	MK378400.1	2018	China
YN01	MK378401.1	2018	China
ZJ01	MK378405.1	2018	China
DSH14	MK092469.1	2018	China
DSH25	MK092470.1	2018	China
DSH27	MK092462.1	2018	China
JX15	MK092471.1	2018	China
JX19	MK092463.1	2018	China
JX20	MK092472.1	2018	China
JX25	MK092464.1	2018	China
JX26	MK092465.1	2018	China
JX30	MK092466.1	2018	China
JX33	MK092467.1	2018	China
JX37	MK092473.1	2018	China
JX38	MK092474.1	2018	China
KA1(1925)	MH447541.1	2018	Korea
KF1(PPV6-1)	MH447535.1	2018	Korea
KF2(2044)	MH447536.1	2018	Korea
KF3(2011)	MH447537.1	2018	Korea
KF4(1940)	MH447538.1	2018	Korea
KF5(405)	MH447539.1	2018	Korea
KF6(403)	MH447540.1	2018	Korea
FJ2017	MG760726.1	2018	China
P1_OK/USA	MW051672.1	2020	USA
