# Supplementary material for: Codon Usage for Genetic Diversity, and Evolutionary Dynamics of Novel Porcine Parvoviruses 2 through 7 (PPV2–PPV7)
Source: Viruses. 2022 Jan 18;14(2):170. doi: 10.3390/v14020170 (PMC8876854; doi:10.3390/v14020170)
Supplement: Supplementary file 1 [file viruses-14-00170-s001.zip › Supplementary Table S6.pdf]

Table S6. Reference strains information of PPV7.

Strsins	GenBank accession NO.	Year	Area
42-NS1 (REP) and capsid (CP) genes	KU563733.1	2016	USA
GX-1	MZ846209.1	2017	China
GX-6	MZ846210.1	2017	China
GX-14	MZ846211.1	2017	China
GX-15	MZ846212.1	2017	China
GX-30	MZ846213.1	2017	China
GD-2014-2	KY996757.1	2017	United Kingdom
GD-2014-3	KY996758.1	2017	United Kingdom
GX2	MG543456.1	2017	China
GX3	MG543457.1	2017	China
GX5	MG543458.1	2017	China
GX6	MG543459.1	2017	China
GX28	MG543460.1	2017	China
GX29	MG543461.1	2017	China
GX30	MG543462.1	2017	China
GX31	MG543463.1	2017	China
GX32	MG543464.1	2017	China
GX34	MG543465.1	2017	China
GX35	MG543466.1	2017	China
GX44	MG543467.1	2017	China
GX45	MG543468.1	2017	China
GX47	MG543470.1	2017	China
GX48	MG543469.1	2017	China
GX49	MG543471.1	2017	China
GX50	MG543472.1	2017	China
FJFZ2017	MG696111.1	2017	China
FJLY2017	MG696112.1	2017	China
JL-5	MZ846217.1	2018	China
JL-13	MZ846218.1	2018	China
JL-71	MZ846223.1	2018	China
SWE2	MG914435.1	2018	China
GZ02	MK378411.1	2018	China
PPV7-55	MK092477.1	2018	China
PPV7-60	MK092478.1	2018	China
PPV7-77	MK092479.1	2018	China
PPV7-80	MK092480.1	2018	China
PPV7-87-clone1	MK092481.1	2018	China

PPV7-87-clone2	MK092482.1	2018	China
DJH11	MK092484.1	2018	China
DJH12	MK092485.1	2018	China
DJH13	MK092486.1	2018	China
DJH14	MK092487.1	2018	China
DJH19	MK092488.1	2018	China
DJH20	MK092489.1	2018	China
DJH23	MK092490.1	2018	China
DJH24	MK092491.1	2018	China
DJH26	MK092492.1	2018	China
JX10	MK092493.1	2018	China
JX15	MK092494.1	2018	China
JX21	MK092495.1	2018	China
JX38	MK092496.1	2018	China
KF1	MH422962.1	2018	Korea
KF2	MH422963.1	2018	Korea
KF3	MH422964.1	2018	Korea
KF4	MH422965.1	2018	Korea
KF5	MH422966.1	2018	Korea
K63	MH422967.1	2018	Korea
LB4	MK092483.1	2018	China
BO.s17.6	MG991309.1	2018	Polska
KU.f12.1-5	MG991310.1	2018	Polska
PB.f11.1-5	MG991311.1	2018	Polska
WT.f11.6-10	MG991312.1	2018	Polska
ZD.f9.1-5	MG991308.1	2018	Polska
AHbz	MK484100.1	2019	China
AHhf	MK484102.1	2019	China
AHmas	MK484101.1	2019	China
P1_OK/USA	MW051671.1	2020	USA
P2_OK/USA	MW051670.1	2020	USA
COL/Cundinamarca7.16/2015	MT747168.1	2020	Colombia
