# Supplementary material for: Codon Usage for Genetic Diversity, and Evolutionary Dynamics of Novel Porcine Parvoviruses 2 through 7 (PPV2–PPV7)
Source: Viruses. 2022 Jan 18;14(2):170. doi: 10.3390/v14020170 (PMC8876854; doi:10.3390/v14020170)
Supplement: Supplementary file 1 [file viruses-14-00170-s001.zip › Supplementary Table S7.pdf]

Table S7. Properties of structural protein genes from PPV strains relative synonymous codon usage analysis in this study (Black font RSCU>1.6, Black font underlined RSCU<0.6) (mean value ± SD).

Categories	PPV2	PPV3	PPV4	PPV5	PPV6	PPV7	ALL
**TTT(Phe)**	0.602 ± 0.052	1.220 ± 0.015	1.210 ± 0.022	1.581 ± 0.016	1.383 ± 0.013	**0.089 ± 0.047**	1.014 ± 0.559
**TTC(Phe)**	1.398 ± 0.052	0.780 ± 0.015	0.791 ± 0.022	**0.419 ± 0.016**	0.617 ± 0.013	1.457 ± 0.047	0.910 ± 0.423
**TTA(Leu)**	**0.100 ± 0.000**	0.787 ± 0.107	**1.749 ± 0.054**	**2.152 ± 0.078**	**0.463 ± 0.041**	**0.100 ± 0.083**	0.892 ± 0.869
**TTG(Leu)**	0.639 ± 0.044	1.519 ± 0.062	0.867 ± 0.107	0.950 ± 0.067	**1.839 ± 0.096**	**0.023 ± 0.063**	0.973 ± 0.644
**CTT(Leu)**	0.900 ± 0.118	1.303 ± 0.067	1.147 ± 0.076	0.885 ± 0.028	1.870 ± 0.100	**0.308 ± 0.037**	1.069 ± 0.519
**CTC(Leu)**	**2.158 ± 0.122**	0.653 ± 0.053	**0.346 ± 0.063**	**0.122 ± 0.004**	0.781 ± 0.099	0.792 ± 0.120	0.809 ± 0.712
**CTA(Leu)**	**0.238 ± 0.050**	**0.597 ± 0.066**	1.162 ± 0.063	1.400 ± 0.077	**0.240 ± 0.002**	1.336 ± 0.235	0.829 ± 0.537
**CTG(Leu)**	**1.959 ± 0.098**	1.140 ± 0.035	0.729 ± 0.057	**0.491 ± 0.031**	0.807 ± 0.082	**2.077 ± 0.194**	1.201 ± 0.668
**ATT(Ile)**	0.761 ± 0.126	1.272 ± 0.043	1.388 ± 0.030	**1.715 ± 0.044**	**2.362 ± 0.045**	**0.005 ± 0.024**	1.250 ± 0.808
**ATC(Ile)**	1.215 ± 0.173	1.110 ± 0.047	**0.464 ± 0.061**	**0.556 ± 0.022**	**0.396 ± 0.041**	**2.082 ± 0.077**	0.970 ± 0.644
**ATA(Ile)**	1.018 ± 0.101	0.613 ± 0.013	1.141 ± 0.064	0.729 ± 0.043	**0.250 ± 0.002**	**0.232 ± 0.076**	0.664 ± 0.379
**GTT(Val)**	**0.411 ± 0.105**	1.153 ± 0.041	**2.002 ± 0.103**	**1.969 ± 0.091**	1.056 ± 0.017	**0.160 ± 0.024**	1.125 ± 0.765
**GTC(Val)**	0.731 ± 0.123	0.764 ± 0.056	**0.014 ± 0.046**	**0.254 ± 0.037**	**0.573 ± 0.017**	1.047 ± 0.102	**0.564 ± 0.374**
**GTA(Val)**	1.138 ± 0.120	0.906 ± 0.056	0.945 ± 0.138	0.996 ± 0.078	**0.240 ± 0.000**	**0.269 ± 0.088**	0.749 ± 0.391
**GTG(Val)**	**1.719 ± 0.095**	1.179 ± 0.039	1.038 ± 0.177	0.784 ± 0.055	**2.122 ± 0.008**	**1.620 ± 0.088**	1.410 ± 0.496
**TCT(Ser)**	1.208 ± 0.082	**1.991 ± 0.115**	**1.905 ± 0.089**	1.273 ± 0.091	**2.372 ± 0.028**	0.120 ± 0.009	1.478 ± 0.800
**TCC(Ser)**	1.077 ± 0.051	0.838 ± 0.079	0.789 ± 0.091	**0.561 ± 0.048**	1.026 ± 0.023	1.089 ± 0.067	0.896 ± 0.207
**TCA(Ser)**	**0.362 ± 0.073**	0.881 ± 0.059	0.798 ± 0.064	**1.622 ± 0.115**	**0.244 ± 0.038**	0.613 ± 0.122	0.753 ± 0.491
**TCG(Ser)**	0.832 ± 0.162	**0.199 ± 0.003**	**0.345 ± 0.057**	**0.203 ± 0.052**	**0.326 ± 0.038**	**0.464 ± 0.147**	**0.395 ± 0.236**
**AGT(Ser)**	**0.506 ± 0.070**	1.596 ± 0.025	0.813 ± 0.055	0.997 ± 0.072	**1.812 ± 0.008**	**0.296 ± 0.125**	1.003 ± 0.598
**AGC(Ser)**	**2.015 ± 0.094**	**0.494 ± 0.007**	1.349 ± 0.073	1.337 ± 0.020	**0.230 ± 0.000**	**2.053 ± 0.172**	1.247 ± 0.756

**CCT(Pro)**	0.707 ± 0.067	1.427 ± 0.028	1.154 ± 0.041	**2.027 ± 0.062**	**2.516 ± 0.027**	<u>**0.425 ± 0.060**</u>	1.376 ± 0.791
**CCC(Pro)**	**1.905 ± 0.039**	1.043 ± 0.020	<u>**0.196 ± 0.036**</u>	<u>**0.115 ± 0.005**</u>	0.630 ± 0.023	0.688 ± 0.074	0.763 ± 0.655
**CCA(Pro)**	<u>**0.564 ± 0.099**</u>	0.798 ± 0.029	**2.020 ± 0.082**	1.371 ± 0.069	0.630 ± 0.002	0.978 ± 0.199	1.060 ± 0.552
**CCG(Pro)**	0.822 ± 0.142	0.722 ± 0.029	0.633 ± 0.110	<u>**0.487 ± 0.078**</u>	<u>**0.233 ± 0.023**</u>	0.998 ± 0.231	0.649 ± 0.267
**ACT(Thr)**	0.643 ± 0.072	1.292 ± 0.031	0.871 ± 0.079	0.679 ± 0.021	**1.615 ± 0.025**	<u>**0.384 ± 0.078**</u>	0.914 ± 0.457
**ACC(Thr)**	**1.755 ± 0.094**	1.121 ± 0.058	0.629 ± 0.041	0.680 ± 0.044	1.120 ± 0.038	0.875 ± 0.145	1.030 ± 0.412
**ACA(Thr)**	<u>**0.460 ± 0.114**</u>	1.079 ± 0.118	**2.170 ± 0.089**	**2.247 ± 0.053**	<u>**0.390 ± 0.035**</u>	0.839 ± 0.249	1.198 ± 0.823
**ACG(Thr)**	1.142 ± 0.111	<u>**0.503 ± 0.068**</u>	<u>**0.329 ± 0.061**</u>	<u>**0.401 ± 0.007**</u>	0.874 ± 0.027	0.990 ± 0.302	0.707 ± 0.339
**GCT(Ala)**	1.150 ± 0.059	**1.640 ± 0.083**	**1.840 ± 0.106**	**1.929 ± 0.085**	**2.304 ± 0.067**	0.670 ± 0.076	1.589 ± 0.588
**GCC(Ala)**	1.545 ± 0.084	1.063 ± 0.107	<u>**0.135 ± 0.047**</u>	<u>**0.408 ± 0.035**</u>	0.723 ± 0.046	0.958 ± 0.114	0.805 ± 0.500
**GCA(Ala)**	0.831 ± 0.068	<u>**0.470 ± 0.040**</u>	**1.770 ± 0.068**	1.215 ± 0.036	<u>**0.422 ± 0.026**</u>	0.697 ± 0.117	0.901 ± 0.513
**GCG(Ala)**	<u>**0.469 ± 0.078**</u>	0.820 ± 0.054	<u>**0.256 ± 0.089**</u>	<u>**0.448 ± 0.087**</u>	<u>**0.557 ± 0.052**</u>	0.766 ± 0.129	<u>**0.553 ± 0.211**</u>
**TAT(Tyr)**	<u>**0.293 ± 0.077**</u>	1.040 ± 0.030	1.305 ± 0.050	1.274 ± 0.035	1.059 ± 0.033	<u>**0.386 ± 0.033**</u>	0.893 ± 0.443
**TAC(Tyr)**	**1.707 ± 0.077**	0.960 ± 0.030	0.695 ± 0.050	0.726 ± 0.035	0.941 ± 0.033	1.160 ± 0.033	1.031 ± 0.372
**CAT(His)**	0.669 ± 0.084	1.404 ± 0.031	1.055 ± 0.048	1.198 ± 0.031	**1.626 ± 0.048**	<u>**0.533 ± 0.070**</u>	1.081 ± 0.421
**CAC(His)**	1.331 ± 0.084	<u>**0.596 ± 0.031**</u>	0.945 ± 0.048	0.802 ± 0.031	<u>**0.374 ± 0.048**</u>	1.013 ± 0.070	0.843 ± 0.335
**CAA(Gln)**	0.813 ± 0.027	0.801 ± 0.037	0.968 ± 0.033	1.501 ± 0.029	1.024 ± 0.062	0.792 ± 0.069	0.983 ± 0.271
**CAG(Gln)**	1.187 ± 0.027	1.199 ± 0.037	1.032 ± 0.033	<u>**0.499 ± 0.029**</u>	0.976 ± 0.062	0.754 ± 0.069	0.941 ± 0.271
**AAT(Asn)**	0.789 ± 0.079	1.167 ± 0.022	1.115 ± 0.019	1.241 ± 0.016	1.594 ± 0.034	<u>**0.095 ± 0.044**</u>	1.000 ± 0.513
**AAC(Asn)**	1.211 ± 0.079	0.833 ± 0.022	0.885 ± 0.019	0.759 ± 0.016	<u>**0.406 ± 0.034**</u>	1.450 ± 0.044	0.924 ± 0.365
**AAA(Lys)**	<u>**0.485 ± 0.053**</u>	1.353 ± 0.033	**1.882 ± 0.046**	**1.814 ± 0.016**	1.160 ± 0.000	1.081 ± 0.138	1.296 ± 0.517
**AAG(Lys)**	1.515 ± 0.053	0.647 ± 0.033	<u>**0.118 ± 0.046**</u>	<u>**0.186 ± 0.016**</u>	0.840 ± 0.000	<u>**0.465 ± 0.138**</u>	0.628 ± 0.513
**GAT(Asp)**	<u>**0.339 ± 0.035**</u>	1.423 ± 0.035	1.429 ± 0.038	1.092 ± 0.020	1.302 ± 0.027	<u>**0.524 ± 0.045**</u>	1.018 ± 0.474
**GAC(Asp)**	**1.661 ± 0.035**	<u>**0.577 ± 0.035**</u>	<u>**0.571 ± 0.038**</u>	0.908 ± 0.020	0.698 ± 0.027	1.022 ± 0.045	0.906 ± 0.412

**GAA(Glu)**	**0.407 ± 0.031**	0.667 ± 0.020	1.533 ± 0.057	1.597 ± 0.014	0.836 ± 0.013	0.660 ± 0.053	0.950 ± 0.496
**GAG(Glu)**	1.593 ± 0.031	1.333 ± 0.020	**0.467 ± 0.057**	**0.403 ± 0.014**	1.164 ± 0.013	0.886 ± 0.053	0.975 ± 0.477
**TGT(Cys)**	**0.328 ± 0.006**	1.330 ± 0.000	1.191 ± 0.052	1.286 ± 0.015	1.422 ± 0.055	**0.220 ± 0.013**	0.963 ± 0.540
**TGC(Cys)**	**1.672 ± 0.006**	0.670 ± 0.000	0.810 ± 0.052	0.714 ± 0.015	**0.578 ± 0.055**	1.326 ± 0.013	0.961 ± 0.437
**CGT(Arg)**	**0.488 ± 0.071**	**0.536 ± 0.049**	**0.295 ± 0.007**	**0.015 ± 0.059**	1.177 ± 0.055	**0.123 ± 0.036**	**0.439 ± 0.414**
**CGC(Arg)**	0.620 ± 0.085	1.436 ± 0.032	**0.153 ± 0.023**	1.160 ± 0.021	1.342 ± 0.071	1.244 ± 0.077	0.992 ± 0.501
**CGA(Arg)**	**0.586 ± 0.110**	**0.443 ± 0.005**	**0.298 ± 0.024**	**0.510 ± 0.128**	**0.390 ± 0.000**	0.942 ± 0.126	**0.528 ± 0.225**
**CGG(Arg)**	0.816 ± 0.094	1.448 ± 0.018	**0.153 ± 0.023**	**0.263 ± 0.080**	0.770 ± 0.059	**0.419 ± 0.114**	0.645 ± 0.475
**AGA(Arg)**	1.471 ± 0.119	1.127 ± 0.029	**4.097 ± 0.129**	**3.812 ± 0.107**	1.550 ± 0.000	**1.615 ± 0.159**	**2.279 ± 1.312**
**AGG(Arg)**	**2.021 ± 0.164**	1.004 ± 0.013	1.006 ± 0.130	**0.232 ± 0.004**	0.762 ± 0.040	**0.292 ± 0.138**	0.886 ± 0.650
**GGT(Gly)**	0.782 ± 0.069	1.192 ± 0.026	1.108 ± 0.080	1.137 ± 0.027	1.439 ± 0.066	**0.354 ± 0.051**	1.002 ± 0.381
**GGC(Gly)**	1.306 ± 0.069	0.826 ± 0.050	**0.161 ± 0.049**	**0.410 ± 0.008**	0.678 ± 0.074	0.976 ± 0.076	0.726 ± 0.408
**GGA(Gly)**	0.976 ± 0.051	0.648 ± 0.026	**2.004 ± 0.070**	**1.834 ± 0.047**	**0.396 ± 0.021**	1.128 ± 0.073	1.164 ± 0.640
**GGG(Gly)**	0.938 ± 0.053	1.334 ± 0.028	0.728 ± 0.080	0.623 ± 0.043	1.487 ± 0.055	0.634 ± 0.062	0.957 ± 0.372
